# The prevalence of pre‐treatment and acquired HIV drug resistance in Vietnam: a nationally representative survey, 2017–2018

**DOI:** 10.1002/jia2.25857

**Published:** 2022-02-23

**Authors:** Vu Quoc Dat, Nguyen Thi Lan Anh, Khuu Van Nghia, Nguyen Thuy Linh, Huynh Hoang Khanh Thu, Tran Thi Minh Tam, Tran Ton, Luong Que Anh, Nguyen Duy Phuc, Phan Thi Thu Huong, Do Thi Nhan, Nguyen Huu Hai, Silvia Bertagnolio, Amy M. Crisp, Seth Inzaule, Natalie E. Dean, Michael R. Jordan, Van Thi Thuy Nguyen

**Affiliations:** ^1^ Department of Infectious Diseases Hanoi Medical University Hanoi Vietnam; ^2^ National Institute of Hygiene and Epidemiology Hanoi Vietnam; ^3^ Pasteur Institute of Ho Chi Minh City Ho Chi Minh City Vietnam; ^4^ Vietnam Administration for HIV/AIDS Control Hanoi Vietnam; ^5^ World Health Organization Geneva Switzerland; ^6^ Department of Biostatistics University of Florida Gainesville Florida USA; ^7^ Division of Geographic Medicine Tufts Medical Center Boston Massachusetts USA; ^8^ Department of Public Health and Community Medicine Tufts University School of Medicine Boston Massachusetts USA; ^9^ Levy Center for Integrated Management of Antimicrobial Resistance (CIMAR) Boston Massachusetts USA; ^10^ World Health Organization Vietnam Country Office Hanoi Vietnam

**Keywords:** HIV, viral suppression, surveillance, acquired HIV drug resistance, pre‐treatment HIV drug resistance, Vietnam

## Abstract

**Introduction:**

Monitoring the population‐level emergence and transmission of HIV drug resistance (HIVDR) is necessary for supporting public health programmes. This study provides a nationally representative prevalence estimate of HIVDR in people initiating antiretroviral therapy (ART) and estimates of acquired HIVDR and viral load (VL) suppression in people who have received it for 12 or ≥48 months in Vietnam.

**Methods:**

The study was conducted between September 2017 and March 2018 following World Health Organization guidance. Thirty ART clinics were randomly sampled using probability proportional to size sampling from a total of 367 ART clinics in the country.

**Results and Discussion:**

In total, 409 patients initiating ART were enrolled into the survey of pre‐treatment HIVDR. The prevalence of any pre‐treatment HIVDR was 5.8% (95% CI 3.4–9.5%), and the prevalence of non‐nucleoside reverse transcriptase inhibitor resistance was 3.4% (95% CI 1.8–6.2%). Four hundred twenty‐nine patients on ART for 12±3 months and 723 patients on ART for ≥48 months were enrolled into the surveys of acquired HIVDR. The prevalence of VL suppression (defined as <1000 copies/ml) in patients on ART for 12±3 and ≥48 months was 95.5% (95% CI 91.3–97.8%) and 96.1% (95% CI 93.2–97.8%), respectively. Among individuals with viral non‐suppression, any HIVDR was detected in 11/14 (weighted prevalence 74.3%) of those on ART for 12±3 months and in 24/27 (weighted prevalence 88.5%) of those receiving ART for ≥48 months.

**Conclusions:**

This nationally representative study of HIVDR found high levels of VL suppression among those on ART for 12 and ≥48 months. Overall, high levels of VL suppression at both time points suggested good adherence among patients receiving ART and quality of treatment services in Vietnam.

**Clinical Trial Number:**

Not applicable

## 1 INTRODUCTION

Antiretroviral therapy (ART) was first introduced in Vietnam in 1996 and is provided free of charge. As of December 2020, there was an estimated 215,000 people living with HIV in Vietnam and about 70% of them were receiving ART [1]. The first nationally representative survey of acquired HIV drug resistance (ADR) in Vietnam was conducted in 2014 among 365 individuals receiving ART for at least 12 months using a cross‐sectional design adopted from contemporary World Health Organiation (WHO) guidance [2, 3]. The prevalence of viral load (VL) suppression (defined as VL <1000 copies/ml) was 95.1% (95% CI 92.3–96.9%), and the prevalence of drug resistance to any antiretroviral (ARV) drug was 4.6% (95% CI 2.8–7.5) [2].

In 2019, WHO reported HIV drug resistance (HIVDR) data from 44 nationally representative HIVDR surveys from 24 low‐ and middle‐income countries, including Vietnam. In this work, we report prevalence estimates of pre‐treatment HIV drug resistance (PDR) among people starting ART and the prevalence of VL suppression and ADR among those taking ART.

## 2 METHODS

### 2.1 Study design and sampling

We conducted a nationally representative cross‐sectional study of PDR and ADR in adults receiving ART for 12±3 months (ADR12) and ≥48 months (ADR48+). At the end of 2016, Vietnam had a total of 367 ART outpatient clinics (OPCs) providing ART to 105,938 adults. Of the 263 OPCs in operation for ≥48 months (representing 92.4% of individuals on ART), 25 were sampled to contribute to the PDR, ADR12 and ADR48+ month surveys. Of the remaining 104 OPCs in operation for <48 months, five OPCs were sampled to contribute to the PDR and ADR12 months study. This sampling approach maximized overlap in sites contributing to each of the three time points [4]. Adults (≥18 years of age) presenting to the sampled clinics were consecutively screened for eligibility and enrolled until the pre‐determined target sample size for each study time point at each site was achieved.

### 2.2 Study population

The PDR survey included people living with HIV (PLHV) presenting to sampled clinics for ART initiation for the first time or re‐initiation of ART if they were on it for less than 1 month and had stopped it for more than 3 months. For ADR surveys, PLHV who have been receiving ART for 12 (±3) months (ADR12) or for ≥48 months were enrolled. All participants in PDR and ADR surveys were 18 years of age or older and provided written informed consent.

### 2.3 Sample size

Sample size calculation followed procedures described by WHO for two‐stage cluster sampling for combined PDR and ADR surveys [3, 5]. The overall sample size was 405 patients for the PDR survey and was 435 and 725 patients for the ADR12 and ADR48+ surveys, respectively.

### 2.4 Survey procedure

All participating OPCs consecutively enrolled eligible patients until reaching the target site‐specific sample size or passing 6 months of the study start date, whichever came first.

Whole blood specimens were collected for plasma VL measurement in both PDR and ADR survey populations. Collecting and processing of specimens followed WHO laboratory guidance [6]. Specimens with VL ≥1000 copies/ml were genotyped. Specimens were tested for HIVDR at one of two laboratories designated by WHO for the purpose of HIVDR surveillance. HIV‐1 RNA was quantified by the Roche Cobas AmpliPrep/Cobas TaqMan HIV‐1 assay (Roche Molecular Systems, Inc., Branchburg, NJ, USA) in both National Institute of Hygiene and Epidemiology and Pasteur Institute in Ho Chi Minh city with a lower limit of quantification of 20 copies/ml. HIVDR genotyping of HIV‐1 reverse transcriptase and protease was performed using standard population sequencing. Sequencing was performed using ABI 3130XL system using the Big‐Dye Terminator v3.1 Cycle Sequencing Kit (Applied Biosystem, Foster City, CA, USA). The Stanford HIV drug resistance algorithm (HIVdb), version 8.4, was used to predict drug resistance and characterize subtypes. Sequences classified as having low‐, intermediate‐ or high‐level HIVDR by Stanford HIVdb were classified as resistant. Sequences classified as potential low‐level resistance or susceptible were classified as susceptible. The outcome “any HIVDR” was defined per WHO guidance as resistance to any nucleoside reverse trascriptase inhibitor (NRTI), efavirenz (EFV) or nevirapine (NVP) and any ritonavir‐boosted PI (PI/r) [7].

### 2.5 Data management and statistical analysis

Data were double entered using Epi Data 3.0 (Odensk, Denmark) and statistical analysis was performed using STATA version 14 (STATA Corp., College Station, TX, USA) following WHO methods. The prevalence of VL suppression and HIVDR were estimated using STATA's survey (SVY) commands [3, 5]. Data were adjusted by number of patients who initiated therapy (PDR), on ART for 12 months (ADR12) or for at least 48 months (ADR48+) in the year prior to the survey initiation, observed clinic‐level patient accrual, number of patients screened and the number of individuals with sequences genotyped. For ADR12 and ADR48+ surveys, the prevalence estimates of HIVDR were further adjusted by clinic‐specific data on retention and clinic‐specific data on unadjusted virological suppression.

### 2.6 Ethics

The study was reviewed and approved by the Institutional Review Board in Biomedical Research, National Institute of Hygiene and Epidemiology, Hanoi, Vietnam (Approval number: IRB‐VN01057‐14/2017).

## 3 RESULTS AND DISCUSSION

Thirty randomly sampled OPCs enrolled patients from September 2017 to March 2018 across 18 of 63 provinces in Vietnam. During the 6 months of the study, the number of eligible patients presenting to OPCs were 1150 across 30 OPCs in the PDR group, 1829 across 30 OPCs in the ADR12 group and 12,199 across 25 OPCs in the ADR48+ group. A total of 1561 patients were enrolled in the survey: 409 patients in the PDR survey, 429 patients in the ADR12 survey and 723 patients in the ADR48+ survey.

Among the 409 people enrolled in the PDR survey, VL was detectable in 393 (96.1%) with 375/409 (91.7%) having VLs >1000 copies/ml. The genotyping success rate was 340/375 (90.7%). A total of 340 genotypes are available from the PDR survey. HIV‐1 subtype CRF01_AE was the predominant subtype and identified in 328/340 (97.1%) patients. Other subtypes included: subtypes B (7/340, 1.9%), subtype C (2/340, 0.7%) and CRF07_BC and CRF25_cp (each of 1% or 0.1%).

In the ADR survey, genotyping was performed only for patients with non‐suppressed VLs (VL >1000 copies/ml). Viral non‐suppression was documented in 18/429 patients (4.2%) receiving ART for 12 months and 32/723 patients (4.4%) receiving ≥48 months.

### 3.1 Pre‐treatment drug resistance

We enrolled 409 patients who presented to OPC for ARV drugs. Participant characteristics are summarized in Table 1. 6.8% (28/409) of patients reported previous exposure to ARV drugs. Of these, 25% (7/28) had received ARV drugs for the prevention of mother‐to‐child transmission of HIV. Advanced HIV disease (defined as WHO Stage 3 or 4) was significantly associated with patients of older age (*p* = 0.013).

**TABLE 1:** Characteristics of participants

	PDR		ADR12		ADR48+	
	*n*	Value	*n*	Value	*n*	Value
Age (median, IQR) (years)	409	33 (28–39)	429	34 (29–42)	723	39 (36–45)
Male (*n*, %)	287	70.2%	290	67.6%	456	63.1%
Geographic region						
North	168/409	41.1%	182/429	42.4%	317/723	43.9%
Central	55/409	13.5%	48/429	11.2%	58/723	8.0%
South	186/409	45.5%	199/429	46.4%	348/723	48.1%
Self‐reported major behaviour risk for HIV infection						
Injection drug use	97/409	23.7%	96/429	22.4%	248/723	34.3%
Unprotected sex with non‐regular partner(s)	191/409	46.7%	219/429	51.0%	418/723	57.8%
Men who have sex with men	76/409	18.6%	57/429	13.3%	9/723	1.2%
CD4 cell count prior to ART initiation						
<100 cells/ml			94/288	32.7%	249/563	44.2%
100 to < 350 cells/ml			101/288	35.1%	263/563	46.7%
≥350 cells/ml			93/288	32.3%	51/563	9.1%
CD4 cell count at time of study enrolment						
<100 cells/ml	43/135	31.9%	10/175	5.7%	11/320	3.4%
100 to < 350 cells/ml	50/135	37.0%	94/175	53.7%	82/320	25.6%
≥350 cells/ml	42/135	31.1%	71/175	40.7%	227/320	70.9%
Viral load						
<1000 copies/ml	33/409	8.1%	411/429	95.8%	691/723	95.6%
1000–5000 copies/ml	17/409	4.2%	2/429	0.5%	9/723	1.2%
>5000 copies/ml	359/409	87.8%	16/429	3.7%	23/723	3.2%
WHO Clinical Stage prior to ART initiation						
Stages 1 and 2	269/373	72.1%	291/412	70.6%	249/661	37.6%
Stages 3 and 4	104/373	27.9%	121/412	29.4%	412/661	62.3%
WHO Clinical Stage at time of study enrolment						
Stages 1 and 2			400/425	93.2%	684/722	94.7%
Stages 3 and 4			25/425	5.8%	38/722	5.3%
Hepatitis B surface antigen (HBsAg)						
HBsAg (+)	25/232	10.8%	32/301	10.6%	67/546	12.3%
HBsAg (–)	207/232	89.2%	269/301	89.4%	479/546	87.7%
Anti‐HCV						
Anti‐HCV (+)	46/205	22.4%	45/278	16.2%	134/485	27.6%
Anti‐HCV (–)	159/205	77.6%	233/278	83.8%	351/485	72.4%
Current ART regimen						
TDF containing regimen	–		422/429	98.4%	484/715	67.7%
ZDV containing regimen	–		7/429	1.6%	231/715	32.3%
EFV containing regimen	–		421/428	98.4%	521/717	72.7%
NVP containing regimen	–		6/428	1.4%	137/717	19.1%
PI/r containing regimen	–		1/428	0.2%	59/717	8.2%
Duration of ART (median, IQR) (months)	–		429	12 (11–14)	723	85 (64–106)

Estimates of PDR are summarized in Table 2. VL was detected in 393/409 (96.1%) specimens. HIVDR genotyping was successful in 340/375 (90.7%) specimens with VL >1000 copies/ml, with any PDR detected in 22/340. After adjusting for the survey design, the nationally representative prevalence of any PDR HIVDR was 5.8% (95% CI 3.4–9.5%) (Table 2).

**TABLE 2:** Prevalence of pre‐treatment HIV drug resistance

	All^a^		ART naïve individuals		Prior ARV drug‐exposed individuals	
Resistance by drug class	*n*/*N*	Prevalence (mean, 95% CI)	*n*/*N*	Prevalence (mean, 95% CI)	*n*/*N*	Prevalence (mean, 95% CI)
Any HIVDR	22/340	5.8% (3.4–9.5%)	16/310	4.6% (2.5–8.4%)	4/20	11.1% (2.9–33.9%)
NNRTI resistance	15/340	3.4% (1.8–6.2%)	10/310	2.7% (1.3–5.5%)	4/20	11.1% (2.9–33.9%)
NRTI resistance	13/340	3.5% (1.8–6.8%)	10/310	2.7% (1.2–6.1%)	2/20	6.5% (1.4–24.7%)
PI resistance	0/333	0% (0.0–1.1%)	0/305	0% (0.0–1.2%)	0/18	0% (0.0–17.6%)
NNRTI+NRTI resistance	6/340	1.2% (0.5–2.8%)	4/310	0.9% (0.3–2.8%)	2/20	6.5% (1.4–24.7%)
NNRTI+NRTI+PI resistance	0/333	0% (0.0–1.1%)	0/305	0% (0.0–1.2%)	0/18	0% (0.0–17.6%)
Resistance by gender						
Women	2/95	2.5% (0.5–11.5%)	2/87	2.7% (0.6–12.3%)	0/7	0% (0.0–35.4%)
Men	20/245	7% (3.9–12.3%)	14/223	5.3% (2.6–10.6%)	4/13	16.6% (3.9–49.1%)
Resistance by age group						
≤25 years old	3/65	6.5% (1.8–20.6%)	3/62	6.8% (1.9–21.9%)	0/2	0% (0.0–65.8%)
>25 years old	19/275	5.6% (3.1–9.9%)	13/248	4.1% (1.9–8.6%)	4/18	12.4% (3–39.6%)
Injection drug use						
Yes	13/77	10.8% (4.5–23.4%)	7/60	6.8% (2.2–19.5%)	4/12	18.2% (3.8–55.5%)
No	9/263	3.5% (1.7–7.1%)	9/250	3.7% (1.8–7.6%)	0/8	0.0% (0.0–32.4%)
Unprotected sex with non‐regular partner(s)						
Yes	9/158	6.3% (3.3–11.6%)	7/146	4.9% (2.4–9.6%)	1/8	6.4% (0.4–56.6%)
No	13/182	5.4% (2.8–10.0%)	9/164	4.4% (2.0–9.4%)	3/12	14.0% (3.4–42.8%)
Men who have sex with men						
Yes	1/72	1.3% (0.2–9.8%)	1/71	1.4% (0.2–10.3%)	0/0	0.0%
No	21/268	6.9% (4.2–11.1%)	15/239	5.5% (3.1–9.7%)	4/20	11.1% (2.9–33.9%)

^a^ There were 10 patients (one female and nine males) with unknown exposure to ARVs.

In specimens with any HIVDR, non‐nucleoside reverse transcriptase inhibitor (NNRTI) resistance was predicted in 15/22 (68.2%); one had NVP resistance, and 14 had NVP and EFV resistance. NRTI resistance was predicted in 13/22 (59.1%), and 6/22 (27.3%) had resistance to both NNRTI and NRTI. The nationally representative prevalence of PDR to the NNRTI drug class was 3.4% (95% CI 1.8–6.2%) (Table 2). We performed genotyping for 333 specimens and no PI resistance was detected in this survey. The prevalence of any PDR was higher in people reporting prior ARV drug exposure compared to those without (20% vs. 5.2%; *p* = 0.02).

Since the introduction of the WHO's PDR survey method in 2014, 12 countries have reported national prevalence estimates of HIVDR with any HIDR prevalence estimates ranging from 8.2% to 23.4% [8, 9, 10, 11]. For Vietnam, this was the first nationally representative survey of PDR using the 2014 WHO method. The prevalence of NNRTI resistance (3.4% [1.8–6.2%]) in this PDR survey was higher than that observed (1.6%, 8/490) in the survey using the WHO's cohort design in four sentinel ART clinics with 501 patients initiating ART from 2009 to 2010 [12]. The prevalence of any PDR in this survey (5.8%, 95% CI 3.4–9.5%) was similar to the overall prevalence of any PDR in Asia as observed in a 10‐year meta‐analysis of 16,088 genotype from the region (from 1996 to 2016), which had increased from 2.6% (95% CI 1.4–4.9) (NNRTI PDR 0.7%, 95% CI 0.2–2.8%) before 2005 to 5.5% (95% CI 3.3–8.6) (NNRTI PDR 4%, 95% CI 2.1–6.7%) in 2014–2016 [12]. However, we reported a lower overall prevalence of PDR than the recent report of 14.7% (95% CI:9.8–21.4%), NNRTI PDR of 9% and PI PDR of 5% in the survey in three ART clinics in HCMC, Vietnam in 2016 [14].

### 3.2 Acquired HIV drug resistance at 12 months after ART initiation (ADR12)

A total of 429 patients from 30 clinics were included in the ADR12 survey. Characteristics of ADR12 survey participants are presented in Table 1. Of 423 patients, 429 (98.4%) initiated first‐line ART, with the majority (99.1%, 419/423) starting tenofovir (TDF) + lamivudine (3TC)+ EFV.

VL suppression and ADR outcomes are summarized in Table 3. VL was < 1000 copies/ml in 411/429 (95.8%); 2/429 (0.5%) had VL from 1000 to 5000 copies/ml, and 16/429 (3.7%) had VL > 5000 copies/ml. After adjusting for the survey design, the nationally representative estimate of VL suppression at 12 months was 95.5% (95% CI 91.3–97.8%) (Table 3).

**TABLE 3:** Prevalence of viral load suppression (< 1000 copies/ml) and HIV drug resistance in individuals receiving ART

	ADR12		ADR48+	
	*n*/*N*	Prevalence % (95% CI)	*n*/*N*	Prevalence % (95% CI)
Prevalence of viral load suppression				
Individuals on ART	411/429	95.5% (91.3–97.8%)	691/723	96.1% (93.2–97.8%)
Individuals on first‐line ART	407/423	96.0% (91.7–98.1%)	658/688	96.2% (93.6–97.8%)
Individuals on first‐line NNRTI‐based ART	407/423	96.0% (91.7–98.1%)	632/658	96.4% (93.6–98.0%)
Individuals on TDF‐based first‐line NNRTI‐based ART	404/421	95.7% (91.4–97.9%)	431/443	97.7% (94.8–99.0%)
Individuals on ZDV‐based first‐line ART	6/7	87.7% (42.3–98.6%)	213/228	93.2% (87.9–96.3%)
Individuals on second‐line ART	4/6	69.0% (30.8–91.7%)	33/35	94.6% (69.6–99.3%)
HIVDR among all individuals				
Any HIVDR	11/429	3.0% (1.6–5.7%)	24/723	3.4% (1.9–6.1%)
NNRTI resistance	11/429	3.0% (1.6–5.7%)	23/723	3.3% (1.8–5.9%)
NRTI resistance	9/429	2.1% (1.0–4.2%)	23/723	3.3% (1.8–5.9%)
PI resistance	0/429	0% (0.0–0.89%)	1/723	0.1% (0.0–0.7%)
HIVDR among individuals on ART with VL ≥1000 cps/ml regimen				
Any resistance	11/14	74.3% (42.8–91.8%)	24/27	88.5% (70.7–96.1%)
NNRTI resistance	11/14	74.3% (42.8–91.8%)	23/27	84.3% (66.0–93.7%)
NRTI resistance	9/14	50.5% (13.1–87.4%)	23/27	84.4% (58.6–95.4%)
PI resistance	0/13	0% (0.0–22.8%)	1/25	2.5% (0.3–21.1%)
NNRTI+NRTI regimen	9/14	50.5% (13.1–87.4%)	22/27	80.2% (56.0–92.8%)
HIVDR among individuals on first‐line ART with VL ≥1000 cps/ml				
Any resistance	9/12	70.7% (39.1–90.0%)	22/25	87.6% (68.9–95.8%)
NNRTI resistance	9/12	70.7% (39.1–90.0%)	21/25	83.1% (64.2–93.1%)
NRTI resistance	8/12	51.1% (10.1–90.6%)	21/25	83.2% (57.6–94.8%)
PI resistance	0/11	0% (0.0–25.9%)	1/24	2.6% (0.3–19.6%)
NNRTI+NRTI resistance	8/12	51.1% (10.1–90.6%)	20/25	78.7% (54.8–91.8%)
HIVDR among individuals on first‐line NNRTI and TDF‐based ART with VL ≥1000 cps/ml				
Any resistance	10/13	72.7% (41.5–90.9%)	8/9	88.0% (55.7–97.7%)
NNRTI resistance	10/13	72.7% (41.5–90.9%)	8/9	88.0% (55.7–97.7%)
TDF resistance	4/13	26.5% (4.8–72.2%)	5/9	53.8% (22.0–82.7%)
ZDV resistance	0/13	0% (0.0–22.8%)	0/9	0% (0.0–29.9%)
FTC/3TC resistance	8/13	47.5% (11.4–86.4%)	7/9	76.1% (31.4–95.7%)
TDF+XTC resistance	4/13	26.5% (4.8–72.2%)	5/9	53.8% (22.0–82.7%)

*Note*: All estimates were weighted for study design (see Methods section).

Abbreviations: 3TC, lamivudine; ART, antiretroviral therapy; FTC, emtricitabine; NNRTI, non‐nucleoside reverse transcriptase inhibitor; NRTI, nucleoside reverse transcriptase inhibitor; TDF, tenofovir; XTC, lamivudine and/or emtricitabine; ZDV, zidovudine.

Out of 14 (14.2%) patients with successful genotyping, 11 had any HIVDR (weighted prevalence 74.3%). The prevalence of any HIVDR among those on ART12±3 months was 3.0% (95% CI 1.6–5.7%). All 11 patients with any HIVDR had NNRTI resistance and nine had NRTI resistance. No PI resistance was observed.

### 3.3 Acquired HIV drug resistance ≥48 months after ART initiation (ADR48+)

A total of 723 patients from 25 clinics were included in the ADR48+ survey (Table 1).

The VL suppression and ADR outcomes are summarized in Table 3. Out of 723 patients, 691 (95.6%) had VL < 1000 copies/ml, 9 (1.2%) had VL > 1000 but ≤ 5000 copies/ml and 23 (3.2%) had VL > 5000 copies/ml. After adjusting for the survey design, the nationally representative estimate of VL suppression was 96.4% (95% CI 93.6–98.0%) in individuals on a first‐line NNRTI‐containing regimen and was 94.6% (95% CI 69.6–99.3%) in individuals receiving second‐line ART.

The prevalence of HIVDR among patients on ART for 12 months (3.0%) was similar to that observed in people receiving ART for at least 48 months (3.4%) (*p* = 0.39 by Fisher's exact test) (Table 1).

This is the second nationally representative ADR survey in Vietnam and we replicated the low prevalence of ADR (3% for ADR12 and 3.4% for ADR48+), which was seen in 2014 among people on ART for at least 36 months (4.6%) [2]. Among countries which completed national ADR surveys between 2014 and 2018, the prevalence of ADR among people on ART for 12 ±3 months ranged from 4.3% to 17.1% and from 4.6% to 28.3% for those receiving ART for ≥48 months [15].

Vietnam has initiated the transition to dolutegravir containing regimens as preferred first‐ and second‐line regimens since 2020 [16]. A third round of HIVDR surveys was conducted in 2020, and the data are being analysed.

Our report has limitations. Firstly, our results were not reported in a timely manner. However, the data were included in the global report and Vietnam has considered these results and decided to continue the first‐line ART regimen containing NNRTIs and keep improving the quality of ART service. Secondly, we were not able to evaluate the HIVDR when the VL was less than 1000 copies/ml. However, we assumed that the prevalence of HIVDR among patient with suppressed VL was low and this issue is recognized as a technical limitation in other studies [17].

## 4 CONCLUSIONS

Vietnam achieved very high level of HIV VL suppression among people on ART and maintained moderate prevalence of HIVDR in patients starting ART and low prevalence of HIVDR in patients receiving ART.

## COMPETING INTERESTS

The authors declare that there are no competing interests.

## AUTHORS’ CONTRIBUTIONS

QD, ND, MRJ, SB and VN developed study protocol, analysed data, interpreted the results and wrote the manuscript. NA, KN, NL, HT, MT, TT, QA, NP, PH, DN and NH contributed to protocol development, monitoring survey implementation and reviewed the manuscript. NA, NL, HT, TT and LQA were involved in the protocol development, performed viral load testing and genotyping and reviewed the manuscript. QD, AC, ND, MRJ and SI performed data analysis. All authors revised the manuscript and approved the final draft.

## FUNDING

This work is supported by the Global Fund to Fight AIDS, Tuberculosis and Malaria and the World Health Organization.

## DISCLAIMER

The views expressed in this paper are those of the authors and do not necessarily represent the official position of the authors’ institutions and World Health Organization.

## Data Availability

The data that support the findings of this study are available from the corresponding author upon reasonable request.
